# Plastics and Food Sources: Dietary Intervention to Reduce BPA and DEHP

**DOI:** 10.1289/ehp.119-a306b

**Published:** 2011-07-01

**Authors:** Kellyn S. Betts

**Affiliations:** Kellyn S. Betts has written about environmental contaminants, hazards, and technology for solving environmental problems for publications including *EHP* and *Environmental Science & Technology* for more than a dozen years.

Purchasing fresh, unpackaged foods and avoiding the use of plastic food processing and storage equipment may help consumers minimize their exposure to bisphenol A (BPA) and bis(2-ethylhexyl) phthalate (DEHP), according to a small but systematic study of associations between diet and people’s levels of these chemicals [*EHP* 119(7):914–920; Rudel et al.].

At the time of this January 2010 study, BPA and DEHP were used widely in food packaging, including cans, plastic wraps, and food storage containers.^1^ Evidence from *in vitro* and animal studies demonstrates BPA’s potential to disrupt endocrine function in a number of organ systems. Animal studies have linked exposure to DEHP to inhibition of testosterone synthesis and adverse effects on the developing male reproductive system. Some epidemiologic evidence also links urinary levels of phthalate metabolites to effects on boys’ reproductive development, male hormone levels, semen quality, and neurobehavioral end points.

Although some scientists believe diet is likely to be a major source of exposure to BPA and DEHP, few empirical data exist to verify this belief. One of the stated goals of the current study was to assess the relative importance of food packaging as a source of exposure to these chemicals. BPA and DEHP are relatively easy to study because they have short biological half-lives and exposure biomarkers that can be measured in urine.

The study involved five 4-person families, including adults and children, who were chosen in part because they reported frequently eating canned foods. Urine samples were collected over 8 days. On days 3–5 family members consumed only food prepared by a caterer who avoided using plastic (including plastic utensils and storage containers) in preparing and packaging the meals and snacks made from fresh ingredients. Families were given stainless steel water bottles and lunch boxes and advised to use only the containers provided. Coffee drinkers were instructed to use a French press or ceramic drip in place of a plastic coffee maker.

When consuming their customary diets, family members’ urine levels of biomarkers for both BPA and DEHP were in the range of the general U.S. population’s, as estimated by the Centers for Disease Control and Prevention’s National Health and Nutrition Examination Survey program. During the time the families were on the fresh-food diet, the geometric mean concentration of BPA dropped by 66%, and mean concentrations of DEHP metabolites decreased by 53–56%.

The researchers note they cannot determine exactly which changes in food sourcing and handling caused the observed exposure changes. In addition, their intervention did not completely eliminate all sources of BPA and DEHP. They state food contamination may occur during premarket processing of whole foods or from the presence of phthalates and BPA in the environment where the food originates.
